# Trends in participant race and sex reporting in lung cancer phase III clinical trials

**DOI:** 10.1002/cnr2.1856

**Published:** 2023-07-08

**Authors:** Faaiq N. Aslam, Rami Manochakian, Yanyan Lou, Gerardo Colon‐Otero, Taimur Sher

**Affiliations:** ^1^ Mayo Clinic Alix School of Medicine Jacksonville Florida USA; ^2^ Mayo Clinic Florida Jacksonville Florida USA

**Keywords:** clinical trials, disparities, lung cancer, NIH Revitalization Act, race, sex

## Abstract

**Background:**

Clinical trials are an essential part of advancing care for cancer patients. Historically, however, racial minorities and females have been underrepresented in these trials. Efforts like the National Institute of Health Revitalization Act attempted to mitigate these disparities, but despite these efforts, they continue to exist. These disparities can subsequently lead to minorities and females receiving suboptimal care.

**Aims:**

The purpose of our study was to understand the changing trends in reporting of participant race and sex as a demographic variable in phase III lung cancer clinical trials published over the last 35 years given these consequences of poor representation.

**Methods and results:**

A total of 426 articles reporting the results of phase III lung cancer clinical trials published from 1984 to 2019 were identified in PubMed. From these articles, data on participant sex and race were collected from the demographic tables to construct the database for this study. This database was subsequently used to determine the rate of reporting of demographic factors like race and sex and the participation trends over the time of minority and female participation in lung cancer phase III clinical trials. The SciPy Stats package for Python was used to calculate descriptive statistics, 95% confidence intervals, two sample *t*‐test, one‐way analysis of variance test, and Pearson's correlation coefficients. The Matplotlib package for Python was used for figure generation. Only 137 (32.2%) of the 426 studies analyzed reported the race of participants. Among those studies, we found that the mean participation rate of White participants was significantly higher (82.65%; *p <* .001). We found a decrease in African American participants and an increase in Asian participants over time. When looking at sex, we found that although the rate of male participation (69.02%) was significantly higher than that of female participation (30.98%), female participation has improved with time at a rate of 0.65% per year.

**Conclusion:**

We found that the reporting and participation of minority races continue to lag that of other demographic factors like sex in phase III clinical trials in lung cancer. Based on our analysis, we note a decline in participation of African Americans in lung cancer phase III clinical trials despite the rising incidence of lung cancer.

## 1 BACKGROUND

Clinical trials are an essential means of improving the outcomes of cancer patients. Racial minorities and females have been underrepresented in clinical trials over time. Historically, such disparities have been a problem of multiracial societies, such as the United States, but with globalization and human migration, health disparities are becoming a global concern. In February 2020, the European Parliament in its report on health inequalities in the European Union identified addressing growing health inequalities as a priority.^1^ The National Institute of Health (NIH) of the United States was tasked to address these growing challenges through the NIH Revitalization Act of 1993. An important mandate of the NIH Revitalization Act was adequate reporting on minority and vulnerable populations in clinical trials. Despite these important efforts, various studies over the ensuing two decades continue to report persistent disparities in clinical trials.^2^, ^3^, ^4^, ^5^

These disparities in the representation of minorities and females in clinical trials can have significant consequences. Given these disparities, it becomes challenging to apply the findings of clinical trials to minority groups as they are not adequately represented in the trials themselves.^6^ Thus, minorities and females may not be receiving the full benefits of scientific advancements as their White and male counterparts.^6^ Another consequence of poor representation is that biological variants seen in minority groups are not adequately studied, creating biases in the definitions of what is “normal” and “abnormal.”^7^ The introduction of these biases is also dangerous as they are propagated into future studies and clinical practice, which can lead to suboptimal care for minority patients.^7^ Thus, it is ever more important to assure clinical trials adequately represent the entire patient population.

As we enter the third decade of the 21st century, with increasing awareness and advancements in communications and global connectivity, an expectation would be that these disparities would be resolved. Given the subsequent consequences associated with such disparities, we set out to evaluate the landscape of race and sex reporting in phase III clinical trials in patients with lung cancer. We selected lung cancer for our study because, as the leading cause of cancer death in the United States, it has represented a significant proportion of phase III cancer studies (14%), and is seen across the entire spectrum of human races and sex.^8^, ^9^, ^10^, ^11^, ^12^, ^13^

The purpose of our study was to determine the historical trends and current landscape of reporting and representation of females and minorities in lung cancer phase III clinical trials. We chose to study lung cancer phase III clinical trials because lung cancer is the most common cause of cancer‐related death and manifests differently depending on one's background; thus, adequate reporting and representation are essential.^8^, ^9^, ^10^, ^11^ Specifically, we looked at participation rates of different races and sex over a 35 years time period (1984–2019). In total, demographic data from 426 lung cancer phase III clinical trials were thoroughly reviewed, and information regarding rates of reporting and participation were obtained and analyzed from these trials. This study is significant because it identifies ongoing disparities in lung cancer phase III clinical trials and draws attention to the poor rates of reporting and representation of minority groups like African Americans despite high incidence rates of lung cancer.

## 2 METHODS

### 2.1 Data collection

To obtain participation data for lung cancer phase III clinical trials, the publically available database, PubMed, was queried using the search terms “((lung[Title] OR pulmonary[Title]) AND (phase 3[Title] OR phase III[Title])) NOT (review) AND (clinicaltrial[Filter] OR randomizedcontrolledtrial[Filter]) AND (fft[Filter])” on April 22, 2020. This search yielded 724 publications, from which 426 publications were analyzed as they met the following inclusion criteria:

1. The study was a phase III lung cancer clinical trial.
2. The study did not present data from a previous clinical trial already recorded in our database (to avoid duplicate recording of data).

**FIGURE 1:**
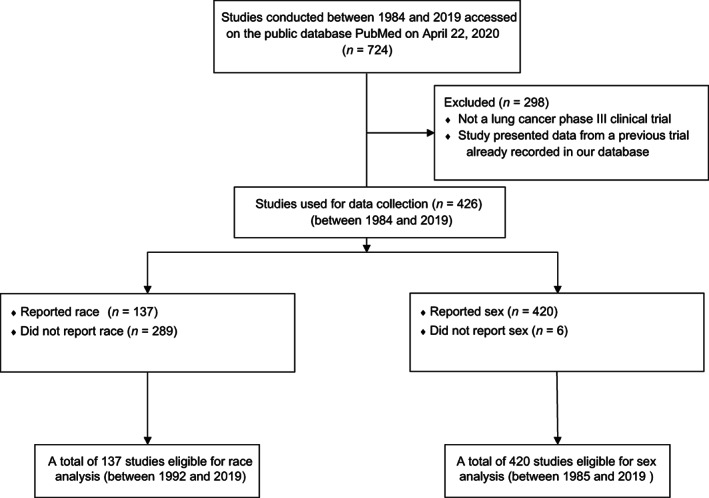
CONSORT diagram illustrating the study selection process.

The 426 publications were subsequently used to collect the year of publication and demographic data relating to participants’ race and sex from the study's demographic table to construct the database for the analysis (Figure 1). Most of the studies the search yielded were multinational studies, which was determined by reviewing the author affiliations of these publications. The review of publically available data could not classify them into discrete categories of the United States versus other regions. This study was considered exempt from Institutional Review Board (IRB) review per institutional policies.

For race, the number of White, Asian, African American, and Hispanic participants was recorded. The category “Other” was also used to include any participants who were listed as Other in the original study or did not belong to the White, Asian, African American, or Hispanic groups. The final category of “Unknown” was used to include any participants who were listed as Unknown in the publication. The number of participants in each category was then converted into the percent of the total participant pool each category represented and was then used to compare participation rates across studies. The studies that did not report demographic data on racial background were annotated as such. For sex, the total number of males and females was recorded. These values were subsequently used to calculate the percent of the participants’ pool that was male or female, and the studies that did not report demographic data regarding sex were annotated as such. The data collected were then used to determine the percentage of studies that reported participant race or sex, differences in the rate of participation among racial groups and between sex, and how participation rates have changed over time.

### 2.2 Statistical analysis

Statistical analysis was done using the SciPy stats package, and figure generation was done using the Matplotlib package for Python.^14^, ^15^ To determine the rate of reporting of race, the percentage of studies that reported race as a demographic and those that did not report race were calculated. Out of the studies that did report race, the percentage of those studies that reported on each racial group was also determined. The same method was also used to determine the rate of reporting of sex.

To determine whether disparities in clinical trials’ participation exist, the mean participation rate of each race and sex across all the studies that reported race or sex was calculated, and the 95% confidence interval (CI) for the mean of each demographic was determined. Then, to compare participation rates among different races, the percent of White participation from each study was compared with the percent participation of the remaining races (Asian, African American, Hispanic, Other, and Unknown) using a two‐sample *t*‐test, and a *p*‐value <.01 was considered statistically significant. A one‐way analysis of variance (ANOVA) test was also used to determine whether a statistically significant difference in participation among the Asian, African American, Hispanic, Other, and Unknown groups exists, and a *p*‐value <.01 was considered statistically significant. To compare participation rates between sex, a two‐sample *t*‐test was used, and a *p*‐value <.01 was considered statistically significant.

Finally, to determine how the participation rate has changed over time, the participation rates of different races from each study were plotted on a scatter plot using the Matplotlib package.^15^ Using those data points, the SciPy stats package was used to calculate Pearson's correlation coefficient and the *p*‐value (with *p <* .05 considered statistically significant), and to draw a line of best fit.^14^ The data points for White participation were plotted with each of the remaining races to serve as a comparison as to how participation rates have changed with time.

## 3 RESULTS

### 3.1 Rates of race reporting and participation trends

**FIGURE 2:**
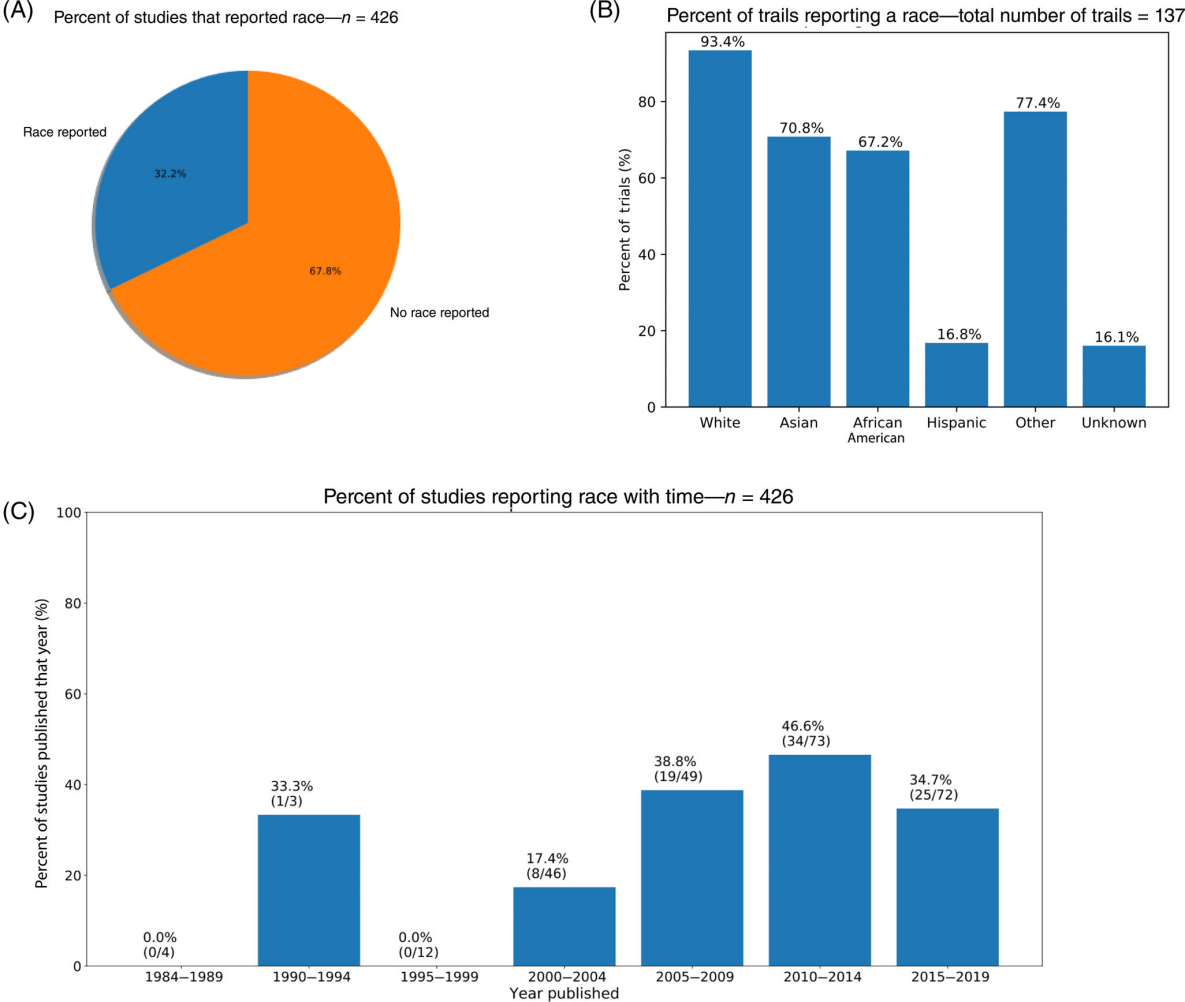
(A) Pie chart illustrating the percentage of studies analyzed that reported race as a patient characteristic. (B) Histogram reporting the percentage of studies (of the studies that reported multiple races) that reported participation rates of each race category. (C) Histogram showing percent of studies published during each 5‐year period that reported demographic data on participant race along with the exact number of studies that reported race over the total number of studies published during that time in our database (as reported in the parentheses).

We analyzed the demographic data from a total of 426 lung cancer phase III clinical trials. We could not identify the United States only studies as most studies had authorships from multiple institutions across various regions in the United States, Europe, and Asia in various combinations. Only 137 studies (32.2%) reported race as a demographic (Figure 2A). The remaining 289 studies (67.8%) did not report race. Interestingly, we found that from all the studies we analyzed, no study published before 1992 reported demographic data on patient race (Figure 2C). After 1992, more studies started reporting race (Figure 2C). However, race reporting did not appear to improve over time since 1993 (Figure 2C). Out of the 137 studies that reported demographic data on participants’ race, 128 studies (93.4%) reported the rate of White participation, 97 studies (70.8%) reported the rate of Asian participation, 92 studies (67.2%) reported the rate of African American participation, 23 studies (16.8%) reported rate of Hispanic participation, 106 studies (77.4%) reported a race that fit into the other category, and 22 studies (16.1%) reported the rate of participants whose race was unknown (Figure 2B).

**FIGURE 3:**
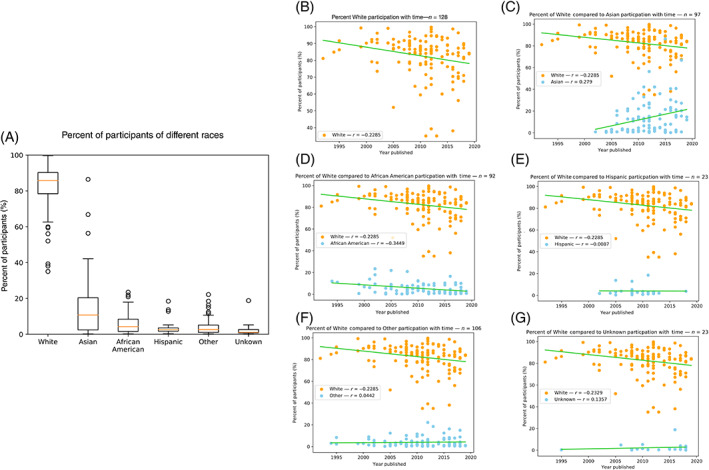
(A) Box plots illustrating the percentage of participants of each race in all the studies analyzed that reported race. (B) A scatter plot with a Pearson correlation coefficient of *r* = −0.23 illustrating how the percentage of White participants has decreased with time. (C) A scatter plot with a Pearson correlation coefficient showing that the percentage of Asian participants has increased with time (*r* = 0.279) and the percentage of White participants has decreased with time (*r* = −0.23). (D) A scatter plot with a Pearson correlation coefficient showing that the percent of African American participants has decreased with time (*r* = −0.34) and the percent of White participants has decreased with time (*r* = −0.23). (E) A scatter plot with a Pearson correlation coefficient showing that the percent of Hispanic participants has not changed with time (*r* = −0.01) and the percent of White participants has decreased with time (*r* = −0.23). (F) A scatter plot with a Pearson correlation coefficient showing that the percent of other participants has not changed with time (*r* = −0.04) and the percent of White participants has decreased with time (*r* = −0.23). (G) A scatter plot with a Pearson correlation coefficient showing that the percent of unknown participants has not changed with time (*r* = 0.14) and the percent of White participants has decreased with time (*r* = −0.23).

The White participants represented 82.55% (95% CI 80.39%–84.71%) of study participants (Table 1; Figure 3A). The mean rates of participation of Asian, African American, Hispanic, Others, and Unknown were 14.03% (95% CI 11.02%–17.04%), 5.53% (95% CI 4.50%–6.56%), 3.97% (95% CI 2.13%–5.81%), 3.99% (95% CI 3.21%–4.77%), and 2.46% (95% CI 0.85%–4.08%), respectively (*p <* .001; Table 1; Figure 3A).

**TABLE 1:** Mean rates of participation of each race across analyzed lung cancer phase III clinical trials.

Race	Number of participants across all studies analyzed	Mean participation rate of each race (%)	95% confidence interval	Pearson's correlation coefficient (change in participation rate with time)	*p*‐Value
White	83 373	82.55	80.39%–84.71%	*m* = −0.51% per year *r* = −0.23, *p <* .01	Two sample *t*‐test (White vs. remaining races) *p <* .001
Asian	13 862	14.03	11.02%–17.04%	*m* = 1.07% per year *r* = 0.279, *p <* .01	One‐way ANOVA test (among remaining races) *p <* .001
African American	3918	5.53	4.50%–6.56%	*m* = −0.30% per year *r* = −0.34, *p <* .001	
Hispanic	446	3.97	2.13%–5.81%	*m* = no change with time *r* = −0.01, *p =* .97	
Other	3711	3.99	3.21%–4.77%	*m* = no change with time *r* = −0.04, *p =* .65	
Unknown	468	2.46	0.85%–4.08%	*m* = no change with time *r* = 0.14, *p =* .55	

Abbreviation: ANOVA, analysis of variance.

When looking at how participation rates changed with time, we found that the rate of White participants decreased at a rate of 0.51% per year (*r* = −0.23, *p <* .01) (Figure 3B). This decrease in participation was accompanied by an increase in the rate of Asian participants by a rate of 1.07% per year (*r* = 0.279, *p <* .01) (Figure 3C). However, the rate of African American participants decreased at a rate of −0.30% per year (*r* = −0.34, *p <* .001) (Figure 3D). The rates of participation among Hispanics (*r* = −0.01, *p =* .97), the other category (*r* = −0.04, *p =* .65), and the unknown category (*r* = 0.14, *p =* .55) did not change with time (Figure 3E–G), although we did not have enough studies that report Hispanic demographics to draw meaningful conclusions about their representation.

### 3.2 Rates of sex reporting and participation trends

**FIGURE 4:**
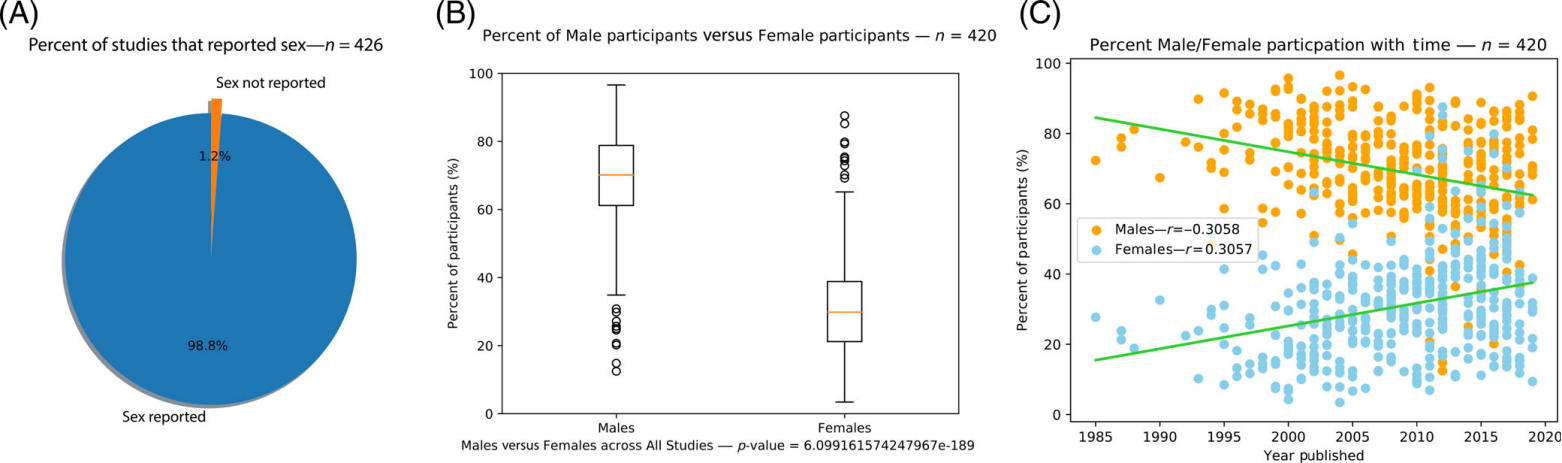
(A) Pie chart illustrating the percentage of studies analyzed that reported sex as a patient characteristic. (B) Box plots illustrating the percentage of participants of each sex in all the studies analyzed that reported sex (*p* < .001). (C) A scatter plot with a Pearson's correlation coefficient showing that the percent of male participants has decreased with time (*r* = −0.31) and the percent of female participants has increased with time (*r* = 0.31).

From the 426 studies that were analyzed, 420 studies (98.8%) reported sex as a patient demographic (Figure 4A). Among those 420 studies, the mean rate of participation for males was 69.02% (95% CI 67.66%–70.38%), and the mean for females was 30.98% (95% CI 29.62%–32.34%), yielding a statistically significant difference in participation rates between males and females (*p <* .001) (Table 2; Figure 4B).

**TABLE 2:** Mean rates of participation of each sex across all analyzed lung cancer phase III clinical trials.

Sex	Number of participants across all studies analyzed	Mean participation rate across all studies (%)	95% confidence interval	*p*‐Value	Pearson's correlation coefficient (change in participation rate with time)	Mean from 1985 to 1995 (%)	Mean from 2015 to 2019 (%)
Male	148 134	69.02	67.66%–70.38%	Two sample *t*‐test (males vs. females)	*m* = −0.65% per year *r* = −0.31, *p <* .001	73.60	65.21
Female	72 685	30.98	29.62%–32.34%	*p <* .001	*m* = 0.65% per year *r* = 0.31, *p <* .001	26.40	34.79

When looking at the change of participation rates over time, we found that the disparity between male and female participation has significantly improved. The rate of male participation has decreased by a rate of 0.65% a year since 1985 (*r* = −0.31, *p <* .001), and the rate of female participation has increased by a rate of 0.65% a year since 1985 (*r* = 0.31, *p <* .001) (Figure 4C). Moreover, we found that the mean rate of participation from 2015 to 2019 for males was 65.21%, and the mean rate of participation for females was 34.79%, which is markedly improved from the mean rate of participation from 1985 to 1995 when the mean participation rate was 73.60% for males and 26.40% for females (Table 2).

## 4 DISCUSSION

Our study is the first one to look at the reporting trends in race and sex in phase III lung cancer trials over the last 35 years. The results of our analysis further substantiate the evidence that continues to highlight the disparities surrounding race in clinical trials.

Examining the trends in reporting sex, we find more positive changes over time. In the studies we reviewed, 98.8% reported participant sex. Analyzing these trends, we noted the progressive increase in women's representation since 1985. While the trends over time are promising, females are still underrepresented with a mean participation rate of 34.97% from 2015 to 2019. This is despite females accounting for 48.9% of lung cancer in the United States.^16^ Similar patterns have also been seen in other, non‐cancer clinical trials as well. For example, cardiovascular and lipid‐lowering agent clinical trials also showed an underrepresentation of females at a participation rate of 33%–38%.^17^, ^18^

We were surprised to find the limited number of studies that reported race before 2000. In reviewing the literature for evidence, we encountered significant variability in race reporting across different studies. As an example, Brahan and Bauchner found significant increases in race reporting from 29.6% to 63.5% in pediatric studies conducted in 1991–1993 and 2000–2002.^19^ In a 2011 study, Geller et al. looked at 86 clinical trials across nine high‐impact journals and found that one‐fifth of the studies failed to report the distribution of participant race.^20^ Unlike the findings from these studies, our findings showed that a significantly larger proportion of lung cancer phase III clinical trials did not report the distribution of participant race. There can be several reasons for such a degree of variance. Pediatric clinical studies, especially pediatric oncology studies, have more centralized review and sponsoring processes and are more likely to be funded by the NIH. Logically, such studies are more likely to comply with the NIH policies including the provisions of the NIH Revitalization Act and, hence, the significant change in the race and sex reporting as noted by Brahan and Bauchner. This increase in race reporting, while encouraging, falls short of the ideal as a third of pediatric oncology studies still did not report race as a variable. Another reason for the lack of reporting on race may be that the majority of the studies are multinational in nature and may include geographical areas with less cultural heterogeneity. This is likely an important limitation of our study. However, the rapid globalization and human migration over the last two decades call for a more acute evaluation. The results of our study in the context of recent demographic changes highlight the significance of addressing this trend as health inequality, and disparities are becoming more of a global issue.

Our findings of minority underrepresentation in lung cancer phase III clinical trials have also been observed in other studies as well. A 2004 study looking at race‐based disparities in cancer clinical trials also found White participants to represent 85.6% of all participants, and another study found White participants to represent 76.3% of all participants in cancer drug clinical trials.^4^, ^21^ One might question the generalizability of this finding in the context of the demographic makeup of the population and the epidemiology of the disease under study. For lung cancer, this is especially important as the incidence in African Americans, the largest minority population in the United States, is 59.5 per 100 000 compared with 61.6 per 100 000 in Whites.^16^ Loree et al. in 2019 evaluated the reporting of race and other demographic variables in the landmark clinical trials leading to the approval of antineoplastic therapies and found a similar finding of a decline in African American participation between 2008 and 2018.^21^

There can be several explanations for this decline in African American participation. One reason can be attributed to historical influences relating to novel research.^6^, ^21^ As a result of studies like the Tuskegee syphilis study, many patients of African American background may be hesitant to participate in novel studies as they do not want to be test subjects.^6^, ^21^ Other factors that may influence African American participation include socioeconomic factors.^21^ According to United States Census data, in 2021, African Americans had the lowest median annual household income at $48 297.^22^ This is in comparison with Asians and Whites who had the highest and second highest median annual household incomes at $101 418 and $77 999, respectively.^22^ As a result of these socioeconomic differences, factors like transportation and access to care may further contribute to lower rates of participation among African American patients.^6^, ^21^

The principles of justice, beneficence, and medicine's professional code of conduct require diversity and inclusion as a mission critical to its social contract with society. These higher moral values call out for all efforts to ensure adequate access of all segments of society to the benefits of biomedical research. While it may be debatable that non‐reporting is not synonymous with not‐being included, it is a strong enough evidence to support the conclusion of non‐inclusion. Although positive changes have been made in the reporting of sex, there is room for improvement when it comes to the reporting of race. The responsibility to move the needle forward falls on the shoulders of all stakeholders. The clinicians and patient care teams should review and discuss the importance of race and sex reporting with the patients in the broader context of its value to society and in addressing the disparities. The clinical trials’ sponsors should work with the study teams to ensure the recording of sex and race among other demographic information in case report forms. Medical publishers should mandate the reporting of race and sex as a criterion for acceptance of publication.

Our study does have limitations. Given that this study was a retrospective analysis, it carries the same weaknesses associated with such studies. As noted in Section 2, we could not reliably decipher, which studies were solely US‐based versus those from other regions as the majority of the manuscripts had author affiliations from various institutions across the globe.

## 5 CONCLUSIONS

In conclusion, we found that disparities in reporting and participation of minority racial groups continue to exist. We believe that requiring reporting of patient demographic data in clinical trials, in compliance with the NIH Revitalization Act, will improve transparency and the rate of reporting. Furthermore, it will critically inform health policymakers and other stakeholders to address the root causes of the underrepresentation of minorities by addressing social barriers and hesitancy to participate in clinical trials.^23^

## AUTHOR CONTRIBUTIONS

**Faaiq N. Aslam:** Conceptualization (equal); data curation (equal); formal analysis (equal); investigation (equal); methodology (equal); visualization (equal); writing – original draft (equal). **Rami Manochakian:** Conceptualization (equal); data curation (equal); formal analysis (equal); methodology (equal); writing – review and editing (equal). **Yanyan Lou:** Conceptualization (equal); data curation (equal); formal analysis (equal); methodology (equal); writing – review and editing (equal). **Gerardo Colon‐Otero:** Conceptualization (equal); data curation (equal); formal analysis (equal); methodology (equal); writing – review and editing (equal). **Taimur Sher:** Conceptualization (equal); data curation (equal); formal analysis (equal); investigation (equal); methodology (equal); visualization (equal); writing – original draft (equal).

## CONFLICT OF INTEREST STATEMENT

The authors have stated explicitly that there are no conflicts of interest in connection with this article.

## ETHICS STATEMENT

Not applicable.

## Data Availability

The datasets used and/or analyzed during the current study are available from the corresponding author on reasonable request.
